# Multifaceted dietary exposures and atrial fibrillation: Bidirectional causal evidence from two-sample Mendelian randomization study

**DOI:** 10.1097/MD.0000000000044722

**Published:** 2025-09-19

**Authors:** Huibin Li, Jialing Xu

**Affiliations:** a Department of Cardiology, The First People’s Hospital of Xiaoshan District, Hangzhou, Zhejiang Province, China; b Department of Oncology, The First People’s Hospital of Xiaoshan District, Hangzhou, Zhejiang Province, China.

**Keywords:** atrial fibrillation, causal relationship, dietary intake, genome-wide association studies, Mendelian randomization

## Abstract

Observational evidence has suggested a link between specific dietary intake and the risk of atrial fibrillation (AF). However, the causal effect and direction between them were still inconclusive. We sought to explore the causal relationship by using two-sample Mendelian randomization (MR). We obtained genome-wide association studies on dietary intake from the UK Biobank. Genome-wide association studies of AF in this study were extracted from the FinnGen consortium. MR estimates were calculated using the inverse-variance weighted method as the main method for analysis. Besides, a series of repeated analyses and sensitivity analyses were performed to assess the robustness of the results. Genetically predicted more pork intake was causally associated with higher risk of AF (odds ratio [OR], 7.185; 95% confidence interval [CI], 1.979–26.086; *P* = .002). Higher salt added to food increased AF risk (OR, 1.465; 95% CI, 1.099–1.9526; *P* = .009). More dried fruit intake was significantly associated with slower progression to AF (OR, 0.501; 95% CI, 0.291–0.864; *P* = .012). There is no convincing evidence for the associations between other dietary intake we studied and the risk of AF. In reverse MR analysis, no evidence was showed that AF could influence the dietary factors in all methods. In this two sample MR analysis, we observed that more pork intake and salt added to food may increase the risk of developing AF. Higher dried fruit intake may be protective against AF. Further investigation of the causal relationships is needed to support our novel findings.

## 1. Introduction

Atrial fibrillation (AF) is a highly prevalent form of cardiac arrhythmia, affecting an estimated 46.3 million individuals worldwide.^[1]^ As human life expectancy increases, the prevalence of AF is anticipated to grow, resulting in rising social and healthcare burdens. Research has shown that AF significantly elevates the risk of conditions such as stroke, dementia, heart failure, and premature death.^[2,3]^ At present, there are no treatments capable of effectively halting or reversing the progression of this condition. Thus, detecting modifiable risk factors associated with AF is crucial for preventive healthcare strategies.

In recent research, lifestyle may have some influence on the development and progression of AF, and the role of diet has attracted considerable attention.^[4]^ Consuming a heart-healthy diet and avoiding a pro-inflammatory diet seemed to lower the individual’s risk of developing AF.^[5]^ Some studies have indicated that excessive intake of meat, such as beef and mutton, is associated with an increased risk of AF,^[6,7]^ while consuming more fish, vegetables, and fruits may reduce the incidence of AF. However, other studies have reported no significant associations between the consumption of coffee, plant-based diets, or oily fish and the risk of AF.^[7–9]^ Evaluating the association between common food intake and AF has significant implications for health management.

Because unknown variables can skew the findings of observational research, it’s hard to confirm that observed links are actually causal.^[10]^ The best way to get around the limits of observation is through randomized controlled trials, but these are often unfeasible due to moral considerations.^[10]^ Mendelian randomization (MR) uses a statistical genetic method to determine if an exposure with a genetic basis can cause a particular clinical result. It accomplishes this by using genetic variations as instrumental variables (IVs).^[11]^ Most of these genetic markers, like single nucleotide polymorphisms (SNPs), are found in large, public genome-wide association studies (GWASs). Precisely because genetic material is randomly allocated at conception, this analytical method can maximally reduce confounding bias and circumvent the risk of reverse causality.^[12,13]^

In this study, a two sample MR analysis was performed using data generated from large GWAS to systematically decipher the causal association of dietary intake on AF risk.

## 2. Materials and methods

### 2.1. Data sources

The SNPs required in the two sample MR analysis should be derived from different studies that are based on a different sample set of the same ethnicity.^[11]^ Summary-level data of the SNPs associated with dietary intake were originated from the MRC Integrative Epidemiology Unit consortium based on the UK Biobank (Table 1). Twelve kinds of dietary intake exposures were included in our study, they are beef intake, lamb intake, pork intake, poultry intake, oily fish intake, nonoily fish intake, dried fruit intake, fresh fruit intake, raw vegetable intake, cooked vegetable intake, coffee intake, and salt added to food. For outcomes, summary statistics data for AF were derived from data in the FinnGen GWAS, which contained 22,068 cases and 116,926 controls. The FinnGen project (https://www.finngen.fi/) initiative seeks to link genetic data with clinical health records to foster advancements in human well-being. The GWASs exclusively involved populations of European descent. Furthermore, because the necessary participant consent and ethical clearances were already in place for those studies, no new approvals were needed for this research.

**Table 1:** Summary information of exposure for dietary intake.

Exposure	GWAS data	Nsnp	Sample	*R* ^2^
Beef intake	ukb-b-2862	8	461,053	3.57 × 10^−5^
Lamb intake	ukb-b-14179	17	460,006	3.56 × 10^−5^
Pork intake	ukb-b-5640	7	460,162	3.32 × 10^−5^
Poultry intake	ukb-b-8006	3	461,900	3.09 × 10^−5^
Oily fish intake	ukb-b-2209	37	460,443	4.02 × 10^−5^
Nonoily fish intake	ukb-b-17627	6	460,880	3.12 × 10^−5^
Dried fruit intake	ukb-b-16576	27	421,764	3.86 × 10^−5^
Fresh fruit intake	ukb-b-3881	33	446,462	4.43 × 10^−5^
Raw vegetable intake	ukb-b-1996	10	435,435	3.04 × 10^−5^
Cooked vegetable intake	ukb-b-8089	9	448,651	3.27 × 10^−5^
Coffee intake	ukb-b-5237	30	428,860	9.25 × 10^−5^
Salt added to food	ukb-b-8121	64	462,630	4.60 × 10^−5^

GWASs = genome-wide association studies, *R*^2^ = phenotype variance explained by genetics, SNPs = single nucleotide polymorphisms.

### 2.2. Study design

**Figure 1.**
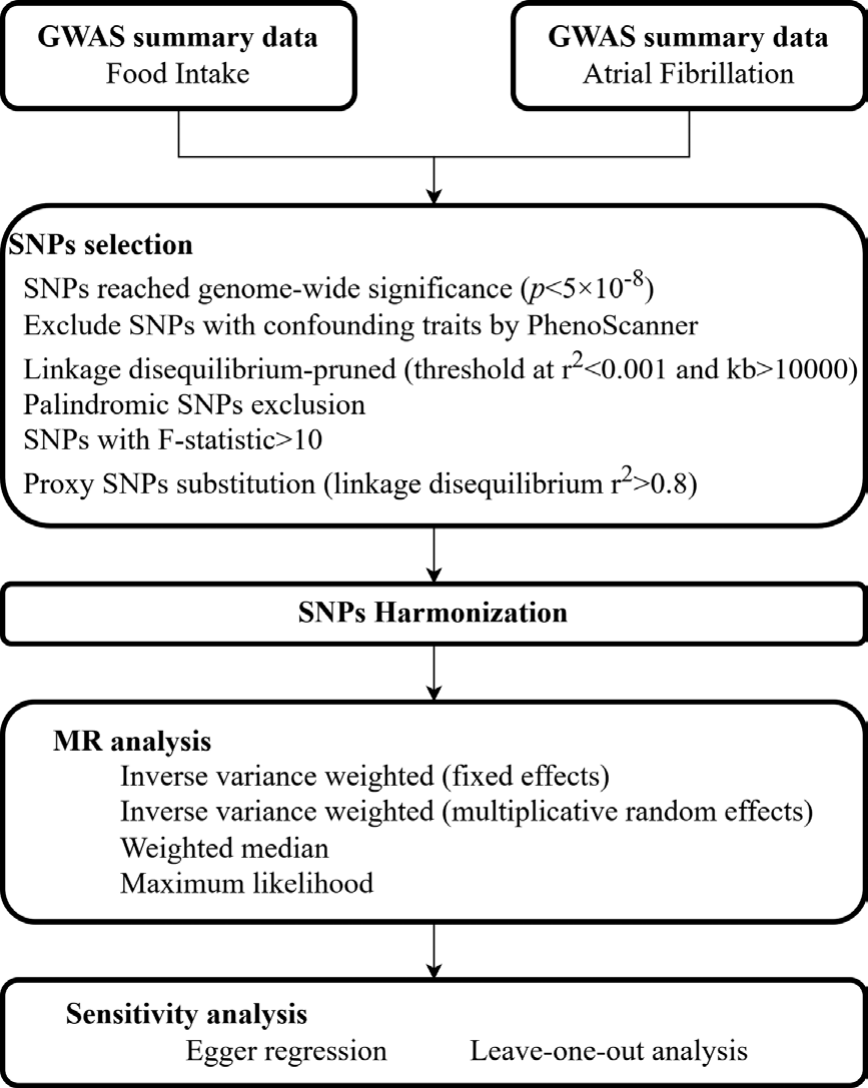
Flow chart of our MR study. GWAS = genome-wide association study, MR = Mendelian randomization, SNPs = single-nucleotide polymorphisms.

**Figure 2.**
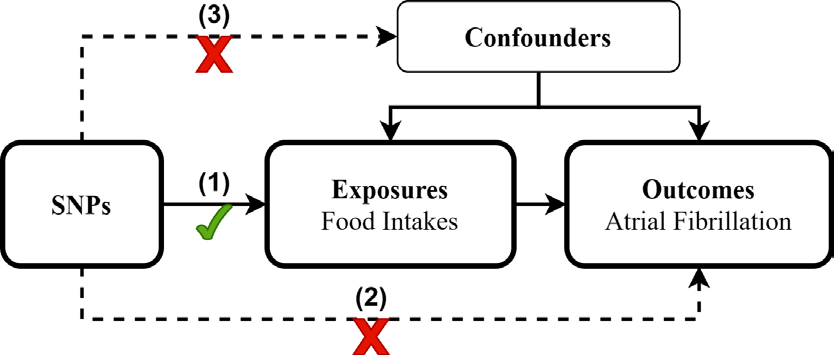
Schematic diagram of the MR analysis in this study. Three assumptions of MR analysis are as follows: SNPs must be associated with dietary intake, SNPs not directly be associated with atrial fibrillation, and SNPs must not be associated with any confounding factor. MR = Mendelian randomization, SNPs = single-nucleotide polymorphisms.

The causal relationships between dietary intake and risk of developing AF were investigated by the two sample MR analysis. SNPs were used as IVs.^[11]^ Subsequently, we further implemented a bidirectional MR approach by designating AF GWAS datasets as exposure variables and dietary intake GWAS datasets as outcome variables, aiming to investigate reciprocal causal relationships through reverse MR analysis. Figure 1 shows a summarization of the research design. The validity of the MR method depends on 3 fundamental conditions: the chosen genetic instruments must exhibit a strong correlation with dietary habits; the genetic variants should not be associated with potential confounding variables; the genetic instruments influence AF exclusively through dietary intake, without other pathways. Figure 2 presents the schematic diagram.

### 2.3. SNP selection and validation

First, to obtain powerful IVs, the SNPs related to dietary intake or AF were extracted from the GWAS dataset at genome-wide significance thresholds (*P* < 5 × 10^−8^). Subsequently, to obtain independent IVs, these SNPs should satisfy linkage disequilibrium (*r*^2^ < 0.001 and clumping window > 10,000 kb).^[14]^ Then, we searched the selected SNPs using LDtrait to identify these SNPs which were possibly related to confounding factors (cardiovascular disease, blood pressure, smoke, alcohol, body mass index, and lipid factors). And for palindromic SNPs, we removed them in case of unexpected biases. Genetic data harmonization between the rest of SNPs and AF was by applying the GWASs on AF. If the SNP could not be found in the AF dataset, a strongly correlated SNP (*r*^2^ > 0.8) was selected as proxy.^[15]^ Lastly, the F statistics were calculated to measure the strength of all the instrument SNPs. F-statistics below the standard threshold of 10 suggest that the IVs have weak estimated effects.^[16]^ All weak IVs will be excluded because of weak instrument bias.

### 2.4. Main analysis

The inverse-variance weighted (IVW) method was the primary analytical approach in the MR analysis. Fixed or random model was used according to heterogeneity. The Weighted median method^[17]^ and maximum likelihood^[18]^ were further applied as complementary analyses for evaluating the causal relationship. The IVW provides MR estimation by calculating the Wald ratio of each SNPs and combining the outcomes in a meta-analysis approach, showing the largest statistical power among all MR methods.^[19]^ Furthermore, scatter plots and forest plots were used to visualize the association between each genetic variant of dietary intake and AF.

In this study, we applied some sensitivity analyses to check robustness of the results. The IVW method and MR Egger regression were used to examine heterogeneity that was assessed by Cochran *Q* test.^[14]^ Egger regression intercepts were used to identify potential horizontal pleiotropy.^[16]^ First, with a *P* value of Cochran *Q* test < .05 indicating the presence of heterogeneity, the outcome of the multiplicative random effects IVW model was adopted as the final results of MR; if not, the fixed effects IVW method was used as the main approach; The MR-Egger regression can detect potential pleiotropy.^[15]^ If the MR-Egger intercept was significantly deviated from 0 (*P* < .05), it was considered to be proof of pleiotropic bias. In addition, a leave-one-out sensitivity analysis was used to identify whether an individual SNP disproportionately affected the overall estimates. Funnel plot is also a method to examine pleiotropy. Symmetric funnel plot is evidence for the absence of potential directional pleiotropy.

After applying Bonferroni correction for 12 multiple comparisons, *P*-values < .004 demonstrate statistical significance, while *P*-values between .004 and .05 indicate nominal suggestive significance.

All the MR analyses were conducted in the software R (version 4.0.3, Vienna, Austria) using the “TwoSampleMR” package (version 0.5.6, Bristol, United Kingdom). The forest plots were drawn in the software R (version 4.0.3 ) using “Forestploter” package (version 0.2.3, Cambridge, United Kingdom).

## 3. Results

### 3.1. Data of exposure and outcome

Overall, we involved 12 kinds of exposures in our study. After a series of quality control measures, the number of SNPs found for each dietary intake varied from 3 to 64 (Table S1, Supplemental Digital Content, https://links.lww.com/MD/Q142). The instrument strength was considered adequate, as all F statistics for the IVs exceeded the value of 10 in our study.

### 3.2. MR analysis

**Figure 3.**
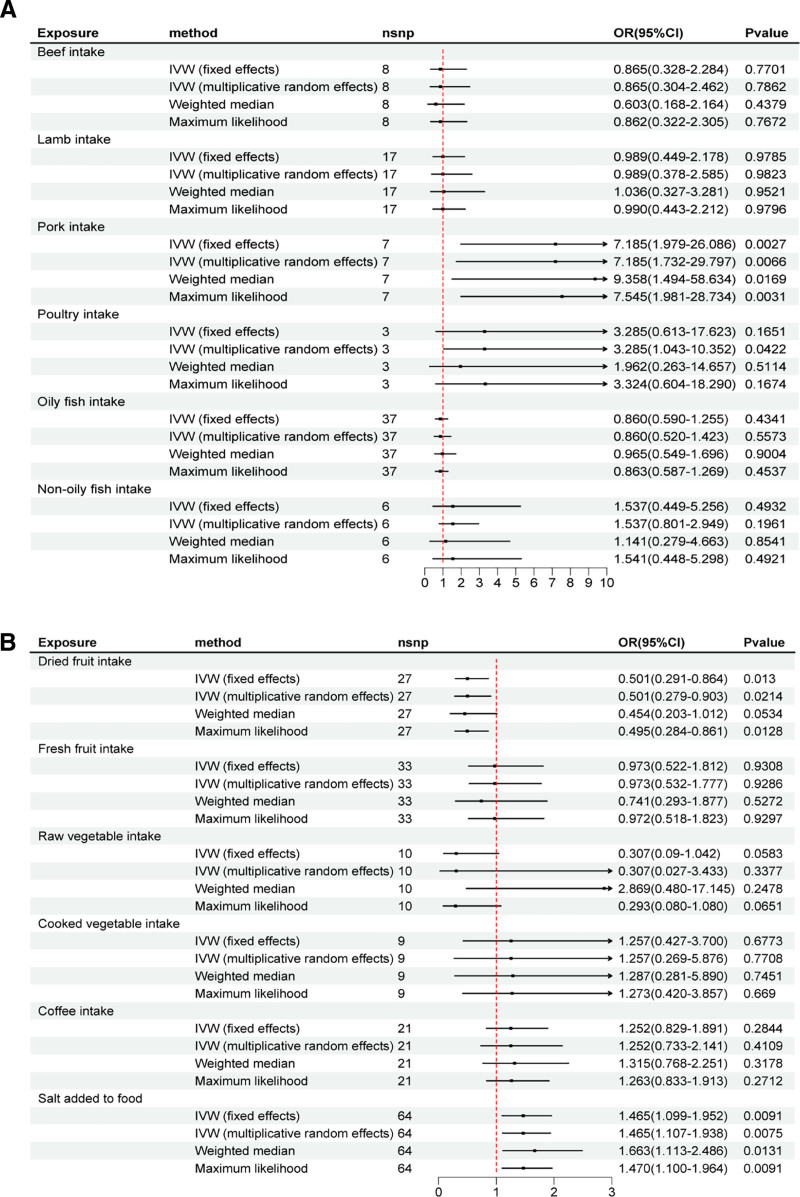
Mendelian randomization estimates of dietary intake on the risk for atrial fibrillation. CI = confidence interval, IVW = inverse-variance weighted, OR = odds ratio, SNPs = Single nucleotide polymorphisms.

Figure 3 presented the consequences of the MR analysis for the association between dietary intake and risk of AF. Causal correlation analysis of dietary intake and AF was conducted by IVW, weight median, and maximum likelihood methods. In the IVW estimates, there is the evidence that increased intake of pork resulted in a higher risk of AF (odds ratio [OR], 7.185; 95% confidence interval [CI], 1.979–26.086; *P* = .002). Some associations showed suggestive significance. Higher salt added to food increased AF risk (OR, 1.465; 95% CI, 1.099–1.9526; *P* = .009). Higher dried fruit intake was associated with a lower incidence of AF (OR, 0.501; 95% CI, 0.291–0.864; *P* = .012). A similar pattern of results was verified by maximum likelihood and weighted median methods (Table S2, Supplemental Digital Content, https://links.lww.com/MD/Q142). On the contrary, no causal relationships were observed between the rest of dietary intake (including beef intake, lamb intake, poultry intake, oily fish intake, nonoily fish intake, fresh fruit intake, raw vegetable intake, cooked vegetable intake, and coffee intake) and AF. There are scatter plots and forest plots depicting the relationship between dietary intake and AF in Figures S1 and S2, Supplemental Digital Content, https://links.lww.com/MD/Q143. Similar results can be observed.

### 3.3. Sensitivity analysis

In order to validate the causal association between food consumption and the risk of developing AF, sensitivity analyses were conducted. Based on the leave-one-out results, it appeared that the overall estimate was remained substantially consistent after each exclusion (Fig. S3, Supplemental Digital Content, https://links.lww.com/MD/Q143). According to the results of Cochran *Q* test, it was revealed that statistically significant heterogeneity effect (*P* < .05) in some MR analyses (Table 2). Consequently, for these analyses random-effects IVW methods were conducted and the results were similar. A significant deviance from 0 of MR-Egger intercept was not observed, suggesting no apparent horizontal pleiotropy (Table 2). Likewise, we lacked evidence of horizontal pleiotropy in our estimations on the basis of the funnel plots (Fig. S4, Supplemental Digital Content, https://links.lww.com/MD/Q143).

**Table 2:** Pleiotropy and heterogeneity test for the associations of dietary intake with atrial fibrillation.

Exposure	Heterogeneity test				Pleiotropy test		
	MR Egger		Inverse-variance weighted		MR Egger		
	Q	Q_pval	Q	Q_pval	Egger_intercept	SE	pval
Beef intake	6.466	3.73E−01	8.128	3.21E−01	0.053	0.043	2.61E−01
Lamb intake	23.382	7.64E−02	23.694	9.64E−02	0.017	0.037	6.61E−01
Pork intake	5.808	3.25E−01	7.302	2.94E−01	−0.101	0.089	3.08E−01
Poultry intake	0.925	3.36E−01	0.934	6.27E−01	−0.046	0.505	9.42E−01
Oily fish intake	62.237	3.09E−03	63.950	2.80E−03	−0.015	0.015	3.33E−01
Nonoily fish intake	1.217	8.75E−01	1.405	9.24E−01	0.018	0.042	6.87E−01
Dried fruit intake	29.955	2.26E−01	30.301	2.55E−01	0.010	0.019	5.95E−01
Fresh fruit intake	27.560	6.44E−01	30.038	5.66E−01	0.016	0.010	1.26E−01
Raw vegetable intake	30.845	1.50E−04	35.098	6.00E−05	0.108	0.103	3.24E−01
Cooked vegetable intake	16.303	2.25E−02	16.326	3.79E−02	−0.014	0.137	9.22E−01
Coffee intake	33.716	1.99E−02	33.882	2.69E−02	−0.003	0.008	7.63E−01
Salt added to food	59.694	5.59E−01	59.903	5.87E−01	−0.003	0.007	6.49E−01

MR = Mendelian randomization, Q = heterogeneity statistic Q, SE = standard error.

### 3.4. Reverse MR analysis

Reverse MR analysis for AF and dietary intake was also conducted by IVW, weight median, and maximum likelihood methods. No evidence was showed that AF could influence the dietary factors in all methods (Table S3, Supplemental Digital Content, https://links.lww.com/MD/Q142).

## 4. Discussion

Previous research has suggested that excessive consumption of red meat is linked to an increased risk of AF,^[6,7]^ whereas higher intake of fish, fruits may be associated with a lower incidence of AF. Other studies did not find a significant link between fruit or fish intake and the risk of AF.^[7–9]^ We conducted a two-sample MR analysis to explore the causal relationships between common dietary items, like coffee, vegetables, fruits, poultry, white meats, red meats, and the risk of AF. The findings indicated that dry fruit consumption may have a protective effect against AF, while increased pork intake and the addition of salt to food were associated with a higher risk of AF. Other dietary factors did not show a statistically significant association with AF risk.

Meat provides an abundant source of protein and essential nutrient such as vitamin B, vitamin A, zinc, and iron. However, meat is also rich in saturated fat and cholesterol. Red meat contains beef, pork, lamb, and so on. A high concentration of myoglobin become the main pigment responsible for its color when in contact with oxygen.^[20,21]^ Poultry is often recommended as one of the healthier substitutes for red meat, which can provide high-quality protein and is often lower in fat. Chicken, turkey, duck, and goose are all good alternatives. It has been reported a positive correlation between red meat consumption and the risk of AF.^[22]^ However, another study found that both red meat and poultry did not increase the risk of AF.^[22]^ In the present study, we found that consuming large amounts of pork may have a higher risk of AF and there are no associations between poultry intake, lamb intake, beef intake, and AF risk. One possible explanation may be that pork consumption often includes fried foods, which tend to contain harmful ingredients such as oxidation products and trans-fatty acids, which are significantly associated with adverse health outcomes.^[23]^ Our MR results should be interpreted cautiously, especially considering the potential pleiotropic bias in MR analysis. Further investigation is warranted to validate the causal relationship between pork intake and AF.

Plant-based diets have also been shown to be effective in managing and reducing AF risk factors. It is possible that inflammation plays a role both in the occurrence and development of AF.^[24]^ Oxidative stress was implicated in the promotion of AF and the maintenance of atrial arrhythmias.^[25]^ The anti-inflammatory and antioxidative components of fruits and vegetables may explain their beneficial role in AF. Based on the IVW method, dried fruit intake might decrease AF risk in our study. However, no association was found between fresh fruit or raw/cooked vegetable intake and AF. Dried fruits are dehydrated into a concentrated form of fresh fruits with the vast majority of nutrients remained. It still conserved many beneficial phytochemicals from the original food by drying fresh fruit.^[26]^ Compared with fresh fruits, dried fruits are favored because of their long shelf life and stability. There’s more fiber and an antioxidant called phenols in dried fruit over fresh, primarily because dried fruits are much more concentrated. The reason why fresh fruits or vegetables are not related to the risk of developing AF needs further study.

Salt, known as sodium chloride, contains about 40% sodium. Dietary sodium is mostly derived from salt. Salts added to food can make the food taste delicious. A study suggested that a high dietary sodium consumption was linked to the higher risk of AF events independently of conventional risk factors.^[27]^ Another prospective study found a U-shaped association between estimated dietary sodium intake and risk of AF among men.^[28]^ Our MR results were similar with the above research. Salt intake can increase the risk of developing AF. It is known that salt intake activates the renin–angiotensin–aldosterone system.^[29,30]^ A rat animal study demonstrated that salt intake caused structural changes in the myocardium by the renin–angiotensin–aldosterone system activation. Rat model studies showed that salt intake was observed to cause myocardial structural alterations by the renin–angiotensin–aldosterone system activation.^[31]^ In 4 different studies, the relative risk of new-onset AF incidence was reduced by 20% to 35% when angiotensin receptor blockades were used.^[32]^ It indicated a connection between the AF and the activation of the renin–angiotensin–aldosterone system.

As for fish, oily fish, and the liver of nonoily fish are all rich in n-3 long-chain polyunsaturated fatty acids. It has been widely noted that polyunsaturated fatty acids may have potential antiarrhythmic properties.^[33]^ But, previous studies about the association between fish intake and the risk of AF had yielded contradictory findings. A U-shaped relationship was found between intake of fatty fish and the risk of AF in the Diet, Cancer, and Health Cohort Study, which included 57,073 patients.^[34]^ However, a Rotterdam Study of 5184 adults (312 who had been diagnosed with AF) did not find any associations between fish intake and risk of AF.^[35]^ Similar results were obtained in the Women’s Health Initiative of 44,720 women (378 who had been diagnosed with AF).^[35]^ Neither the Cohort of Swedish Men nor the Swedish Mammography Cohort supported an advantageous influence of fatty fish or n-3 polyunsaturated fatty acids intake on incident AF.^[8]^ Our MR analysis suggested that neither oily fish intake nor nonoily fish intake was related to the risk of developing AF. It should be noted that, the cooking method of the fish may also influence health outcomes; for example, Mozaffarian et al reported that consuming tuna or other baked, steamed fish, but not fried fish or fish sandwiches, was associated with a lower incidence of AF.^[6]^

Coffee is a complex drink containing numerous substances with biological activity that may affect health.^[36]^ Clinically, caffeine, a constituent of coffee, has been thought to be the primary environmental factor for irregular heartbeats.^[37]^ An early study using a canine model revealed that coffee might cause arrhythmias.^[38]^ According to a case-control study, coffee consumption was significantly associated with an increased risk of acute AF.^[36]^ Yet, latest studies did not think that coffee had an impact on arrhythmias. In a meta-analysis of 6 prospective cohort studies, no association between self-reported coffee consumption and risk of AF was observed.^[39]^ It appeared that caffeine’s influence varied in habitual versus nonhabitual consumers and also depended on the dose, the preparation method, and the intake pattern. Based on our MR study, there was no evidence that coffee consumption could increase the incidence of AF, which was in accordance with the previous MR studies using different database.^[40]^

As far as we know, our study is the first MR study to estimate the causal relations between a wide range of dietary intake and AF comprehensively. The limitations of conventional epidemiological study designs including confounding and reverse causality can be overcome by MR. To enhance the robustness of our findings, we repeated the analysis using multiple methods. Sensitivity analysis was applied to ensure the consistency of causal relationships. Some limitations in this study were also worth acknowledging. First, this study only concentrated on European-ancestry populations, making it more difficult to generalize our results to other ethnicities. Second, horizontal pleiotropy, as an inherent limitation of MR analysis, is difficult to avoid although potential pleiotropic SNPs were discarded and sensitivity analysis showed little horizontal pleiotropy. Third, our study was on the basis of GWAS summary statistics. No GWAS on food preparation methods was found, leading to the difficulty in deducing different effects of the preparation methods of food on the causal association between dietary intake and AF. It still needs further investigation. Finally, the MR analysis can only suggest a potential causal association but cannot provide evidence for specific mechanisms behind the effect of dietary intake on AF.

In conclusion, our two sample MR analysis provides genetic evidence that higher pork intake and higher salt added to food were significantly associated with the increased risk of AF, and that dried fruit intake may be protective against AF. Further research of these novel findings and the potential mechanism underlying specific dietary intake are expected to be explored.

## Acknowledgments

All authors thank the researchers and study participants for their contributions to the GWASs, R packages and datasets used in study, and acknowledge the helpful comments on this paper received from our reviewers and editors.

## Author contributions

**Conceptualization:** Huibin Li, Jialing Xu.

**Data curation:** Huibin Li.

**Investigation:** Huibin Li.

**Methodology:** Jialing Xu.

**Project administration:** Jialing Xu.

**Software:** Jialing Xu.

**Supervision:** Jialing Xu.

**Writing – original draft:** Huibin Li, Jialing Xu.

## Supplementary Material


