# Supplementary material for: Multifaceted dietary exposures and atrial fibrillation: Bidirectional causal evidence from two-sample Mendelian randomization study
Source: Medicine (Baltimore). 2025 Sep 19;104(38):e44722. doi: 10.1097/MD.0000000000044722 (PMC12459541; doi:10.1097/MD.0000000000044722)
Supplement: Supplementary file 2 [file medi-104-e44722-s002.pdf]

Supplementary Figures

Supplementary Figure 1 Scatter plot of SNPs associated with dietary intake and risk on atrial fibrillation. (A) beef intake, (B) lamb intake, (C) pork intake, (D) poultry intake, (E) oily fish intake, (F) non-oily fish intake, (G)dried fruit intake, (H) fresh fruit intake, (I) raw vegetable intake, (J) cooked vegetable intake, (K) coffee intake, (L) salt added to food.

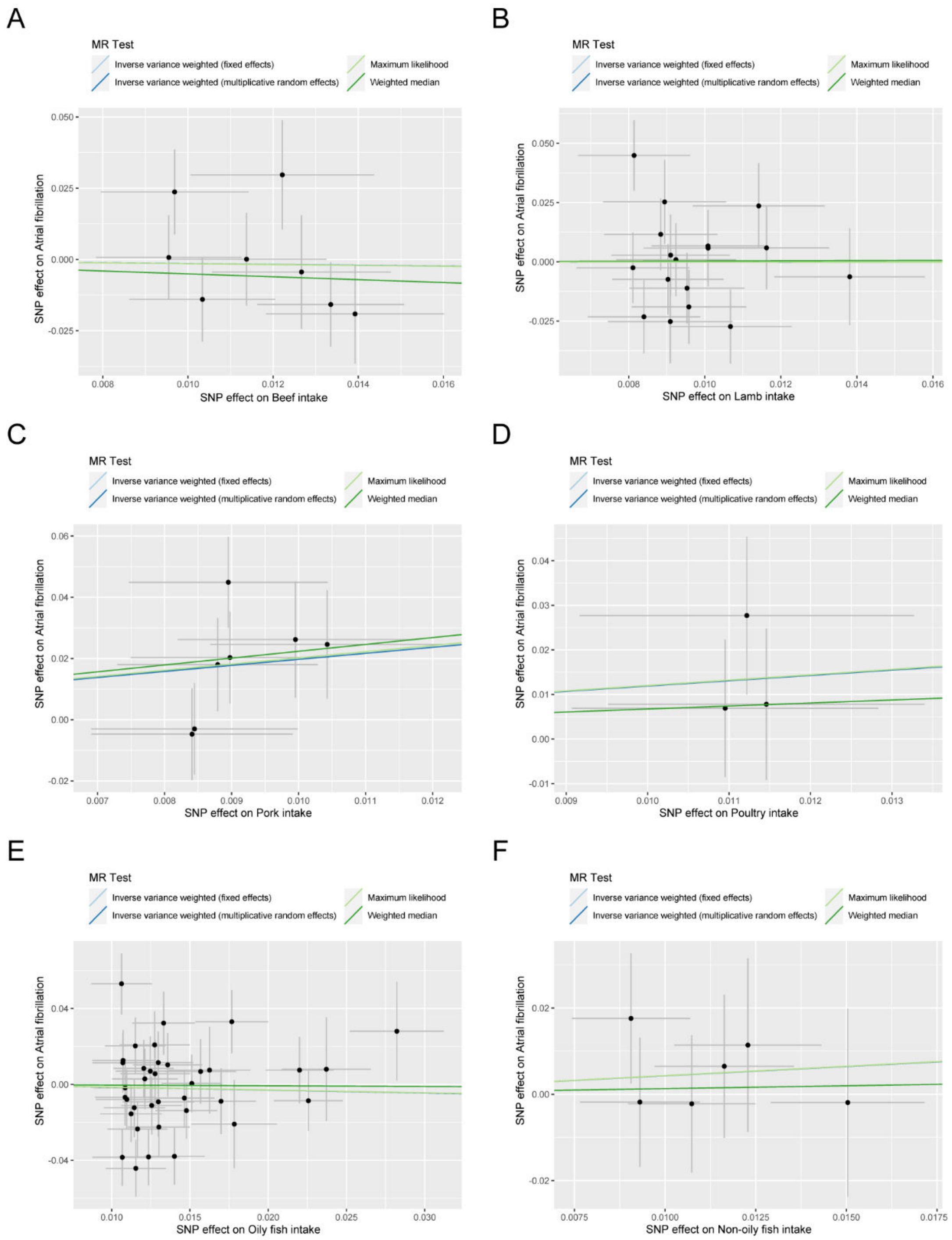

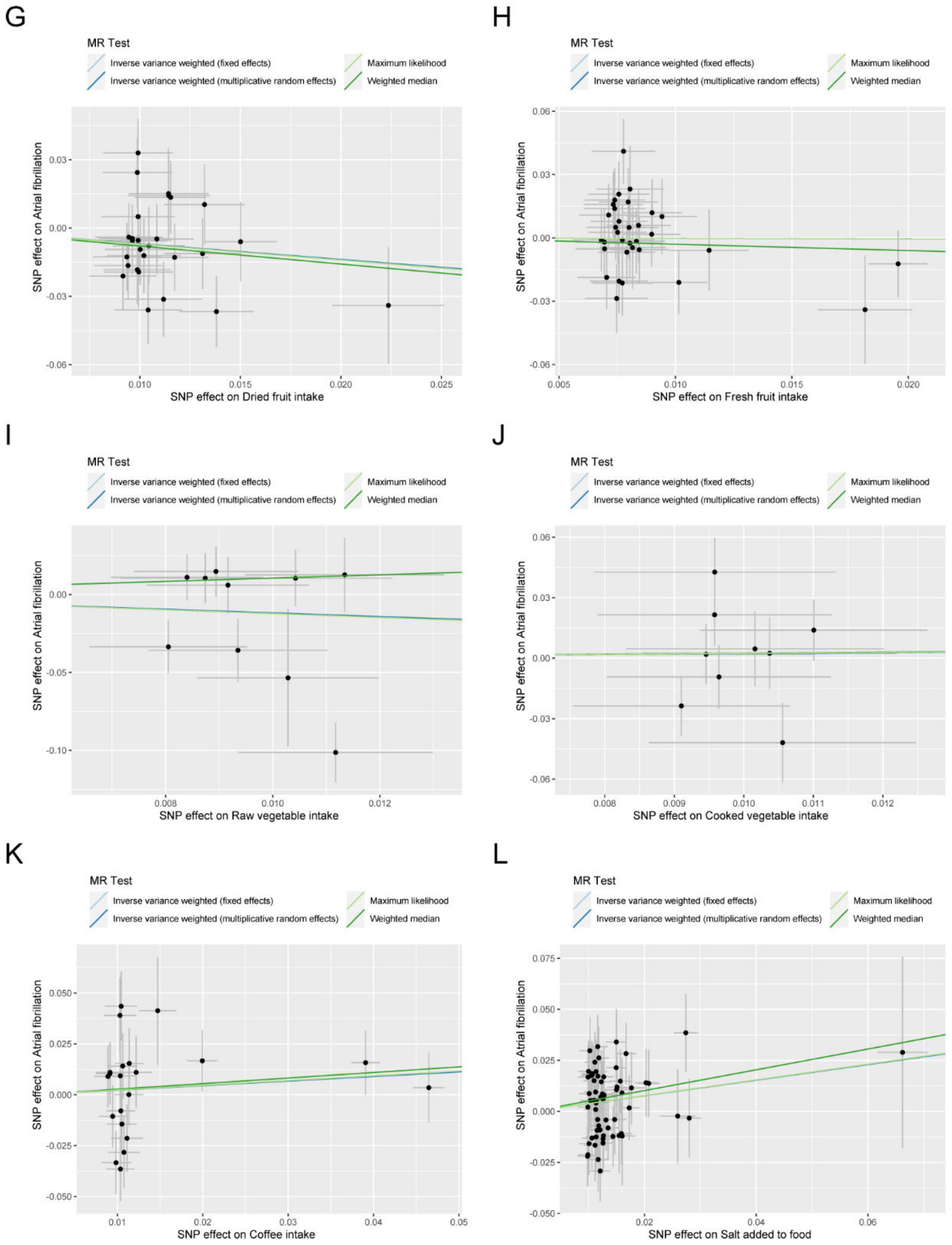
G
MR Test
Inverse variance weighted (fixed effects)
Inverse variance weighted (multiplicative random effects)
Maximum likelihood
Weighted median
SNP effect on Atrial fibrillation
SNP effect on Dried fruit intake
H
MR Test
Inverse variance weighted (fixed effects)
Inverse variance weighted (multiplicative random effects)
Maximum likelihood
Weighted median
SNP effect on Atrial fibrillation
SNP effect on Fresh fruit intake
I
MR Test
Inverse variance weighted (fixed effects)
Inverse variance weighted (multiplicative random effects)
Maximum likelihood
Weighted median
SNP effect on Atrial fibrillation
SNP effect on Raw vegetable intake
J
MR Test
Inverse variance weighted (fixed effects)
Inverse variance weighted (multiplicative random effects)
Maximum likelihood
Weighted median
SNP effect on Atrial fibrillation
SNP effect on Cooked vegetable intake
K
MR Test
Inverse variance weighted (fixed effects)
Inverse variance weighted (multiplicative random effects)
Maximum likelihood
Weighted median
SNP effect on Atrial fibrillation
SNP effect on Coffee intake
L
MR Test
Inverse variance weighted (fixed effects)
Inverse variance weighted (multiplicative random effects)
Maximum likelihood
Weighted median
SNP effect on Atrial fibrillation
SNP effect on Salt added to food

Supplementary Figures

Supplementary Figure 2 Forest plots of SNPs associated with dietary intake and risk on atrial fibrillation. **(A)** beef intake, **(B)** lamb intake, **(C)** pork intake, **(D)** poultry intake, **(E)** oily fish intake, **(F)** non-oily fish intake, **(G)**dried fruit intake, **(H)** fresh fruit intake, **(I)** raw vegetable intake, **(J)** cooked vegetable intake, **(K)** coffee intake, **(L)** salt added to food.

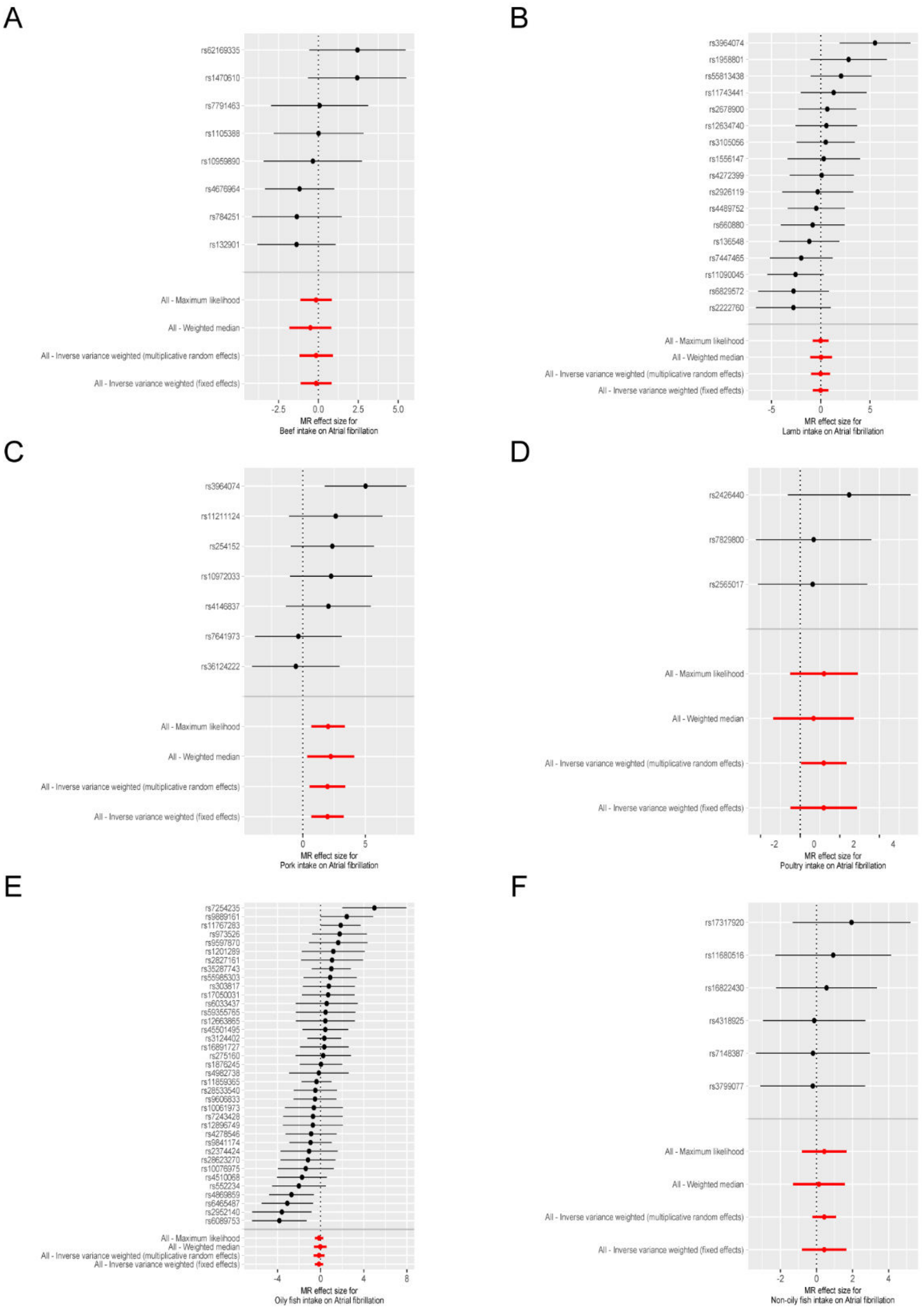

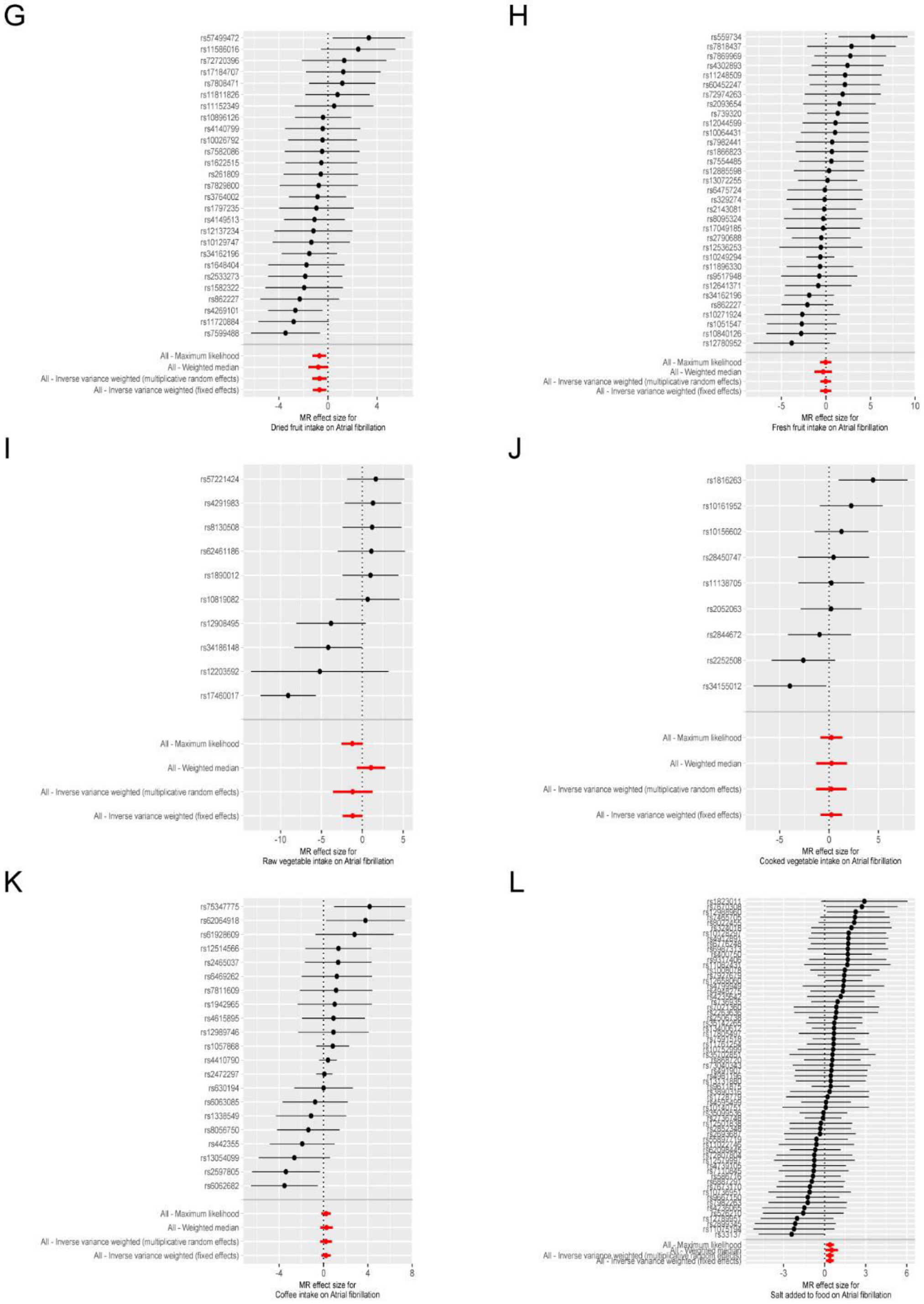

G
rs57499472
rs11586016
rs72720396
rs17184707
rs7808471
rs11811826
rs11152349
rs10896126
rs4140799
rs10026792
rs7582086
rs1622515
rs261809
rs7829800
rs3764002
rs1797235
rs4149513
rs12137234
rs10129747
rs34162196
rs1648404
rs2533273
rs1582322
rs862227
rs4269101
rs11720884
rs7599488
All - Maximum likelihood
All - Weighted median
All - Inverse variance weighted (multiplicative random effects)
All - Inverse variance weighted (fixed effects)
-4
0
4
MR effect size for
Dried fruit intake on Atrial fibrillation
H
rs559734
rs7818437
rs7869969
rs4302893
rs11248509
rs60452247
rs72974263
rs2093654
rs739320
rs12044599
rs10064431
rs7982441
rs1866823
rs7554485
rs12885598
rs13072255
rs6475724
rs329274
rs2143081
rs8095324
rs17049185
rs2790688
rs12536253
rs10249294
rs11896330
rs9517948
rs12641371
rs34162196
rs862227
rs10271924
rs1051547
rs10840126
rs12780952
All - Maximum likelihood
All - Weighted median
All - Inverse variance weighted (multiplicative random effects)
All - Inverse variance weighted (fixed effects)
-5
0
5
10
MR effect size for
Fresh fruit intake on Atrial fibrillation
I
rs57221424
rs4291983
rs8130508
rs62461186
rs1890012
rs10819082
rs12908495
rs34186148
rs12203592
rs17460017
All - Maximum likelihood
All - Weighted median
All - Inverse variance weighted (multiplicative random effects)
All - Inverse variance weighted (fixed effects)
-10
-5
0
5
MR effect size for
Raw vegetable intake on Atrial fibrillation
J
rs1816263
rs10161952
rs10156602
rs28450747
rs11138705
rs2052063
rs2844672
rs2252508
rs34155012
All - Maximum likelihood
All - Weighted median
All - Inverse variance weighted (multiplicative random effects)
All - Inverse variance weighted (fixed effects)
-5
0
5
MR effect size for
Cooked vegetable intake on Atrial fibrillation
K
rs75347775
rs62064918
rs61928609
rs12514566
rs2465037
rs6469262
rs7811609
rs1942965
rs4615895
rs12989746
rs1057868
rs4410790
rs2472297
rs630194
rs6063085
rs1338549
rs8056750
rs442355
rs13054099
rs2597805
rs6062682
All - Maximum likelihood
All - Weighted median
All - Inverse variance weighted (multiplicative random effects)
All - Inverse variance weighted (fixed effects)
-4
0
4
8
MR effect size for
Coffee intake on Atrial fibrillation
L
rs1823011
All - Maximum likelihood
-3
0
3
6
MR effect size for
Salt added to food on Atrial fibrillation

Supplementary Figures

Supplementary Figure 3 Leave-one-out analysis result of SNPs associated with dietary intake and risk on atrial fibrillation. **(A)** beef intake, **(B)** lamb intake, **(C)** pork intake, **(D)** poultry intake, **(E)** oily fish intake, **(F)** non-oily fish intake, **(G)**dried fruit intake, **(H)** fresh fruit intake, **(I)** raw vegetable intake, **(J)** cooked vegetable intake, **(K)** coffee intake, **(L)** salt added to food.

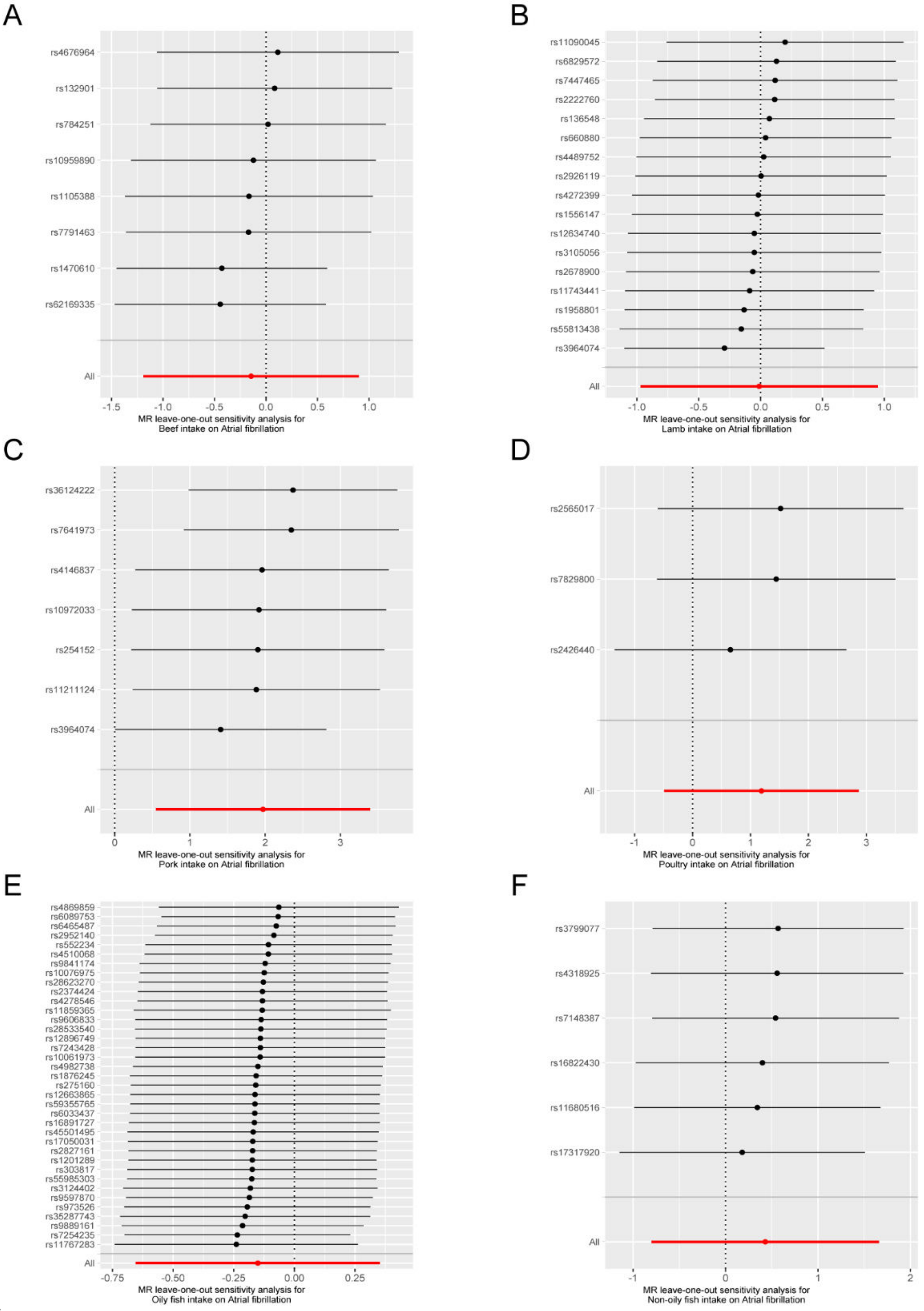

.

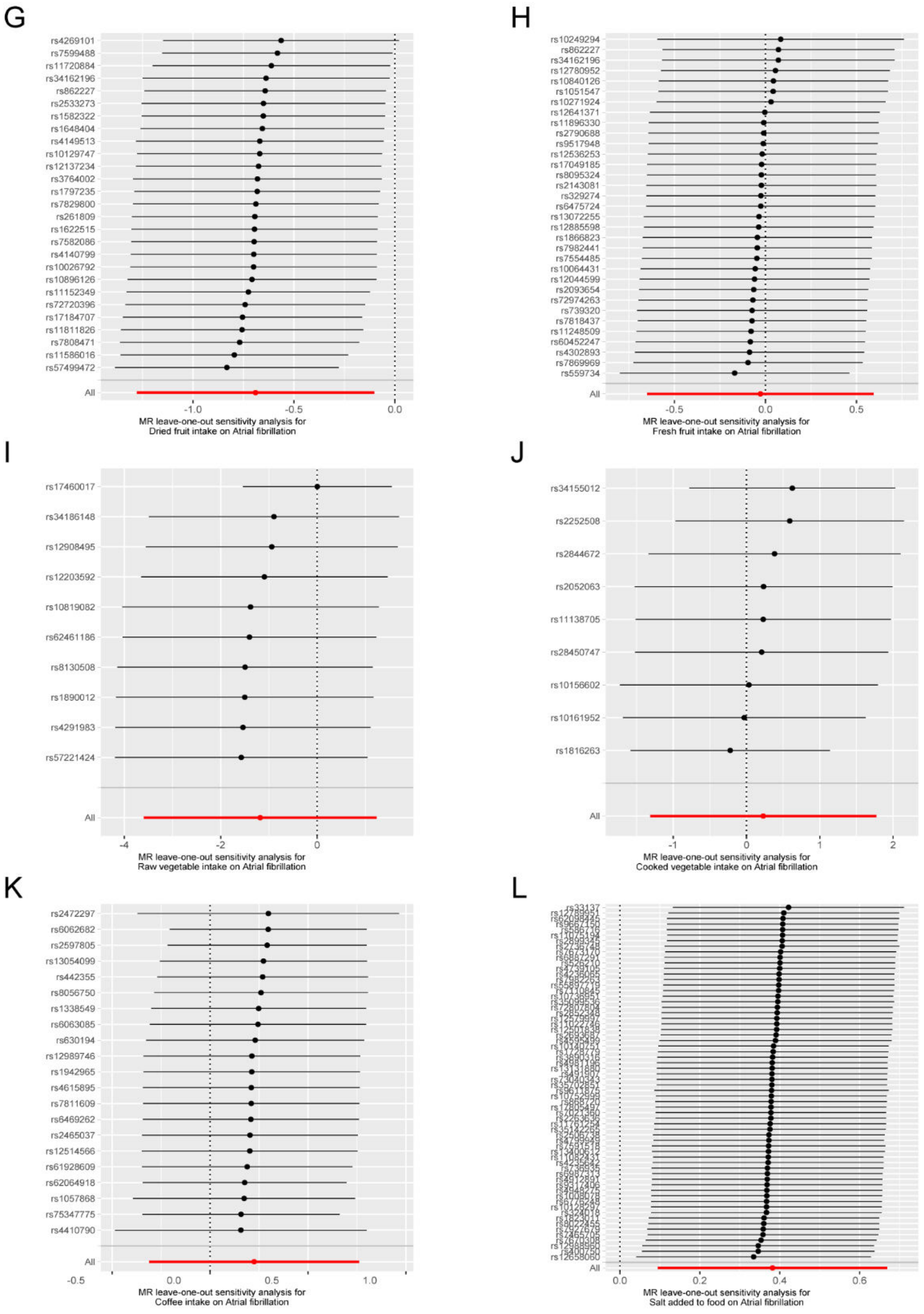
G
rs4269101
rs7599488
rs11720884
rs34162196
rs862227
rs2533273
rs1582322
rs1648404
rs4149513
rs10129747
rs12137234
rs3764002
rs1797235
rs7829800
rs261809
rs1622515
rs7582086
rs4140799
rs10026792
rs10896126
rs11152349
rs72720396
rs17184707
rs11811826
rs7808471
rs11586016
rs57499472
All
-1.0
-0.5
0.0
MR leave-one-out sensitivity analysis for
Dried fruit intake on Atrial fibrillation
H
rs10249294
rs862227
rs34162196
rs12780952
rs10840126
rs1051547
rs10271924
rs12641371
rs11896330
rs2790688
rs9517948
rs12536253
rs17049185
rs8095324
rs2143081
rs329274
rs6475724
rs13072255
rs12885598
rs1866823
rs7982441
rs7554485
rs10064431
rs12044599
rs2093654
rs72974263
rs739320
rs7818437
rs11248509
rs60452247
rs4302893
rs7869969
rs559734
All
-0.5
0.0
0.5
MR leave-one-out sensitivity analysis for
Fresh fruit intake on Atrial fibrillation
I
rs17460017
rs34186148
rs12908495
rs12203592
rs10819082
rs62461186
rs8130508
rs1890012
rs4291983
rs57221424
All
-4
-2
0
MR leave-one-out sensitivity analysis for
Raw vegetable intake on Atrial fibrillation
J
rs34155012
rs2252508
rs2844672
rs2052063
rs11138705
rs28450747
rs10156602
rs10161952
rs1816263
All
-1
0
1
2
MR leave-one-out sensitivity analysis for
Cooked vegetable intake on Atrial fibrillation
K
rs2472297
rs6062682
rs2597805
rs13054099
rs442355
rs8056750
rs1338549
rs6063085
rs630194
rs12989746
rs1942965
rs4615895
rs7811609
rs6469262
rs2465037
rs12514566
rs61928609
rs62064918
rs1057868
rs75347775
rs4410790
All
-0.5
0.0
0.5
1.0
MR leave-one-out sensitivity analysis for
Coffee intake on Atrial fibrillation
L
rs33137
rs12658060
All
0.0
0.2
0.4
0.6
MR leave-one-out sensitivity analysis for
Salt added to food on Atrial fibrillation

Supplementary Figures

Supplementary Figure 4 Funnel plot of the relationship between the causal effect of dietary intake on atrial fibrillation and the reciprocal of the standard deviation of the causal estimation using a single SNP as an instrument. **(A)** beef intake, **(B)** lamb intake, **(C)** pork intake, **(D)** poultry intake, **(E)** oily fish intake, **(F)** non-oily fish intake, **(G)**dried fruit intake, **(H)** fresh fruit intake, **(I)** raw vegetable intake, **(J)** cooked vegetable intake, **(K)** coffee intake, **(L)** salt added to food.

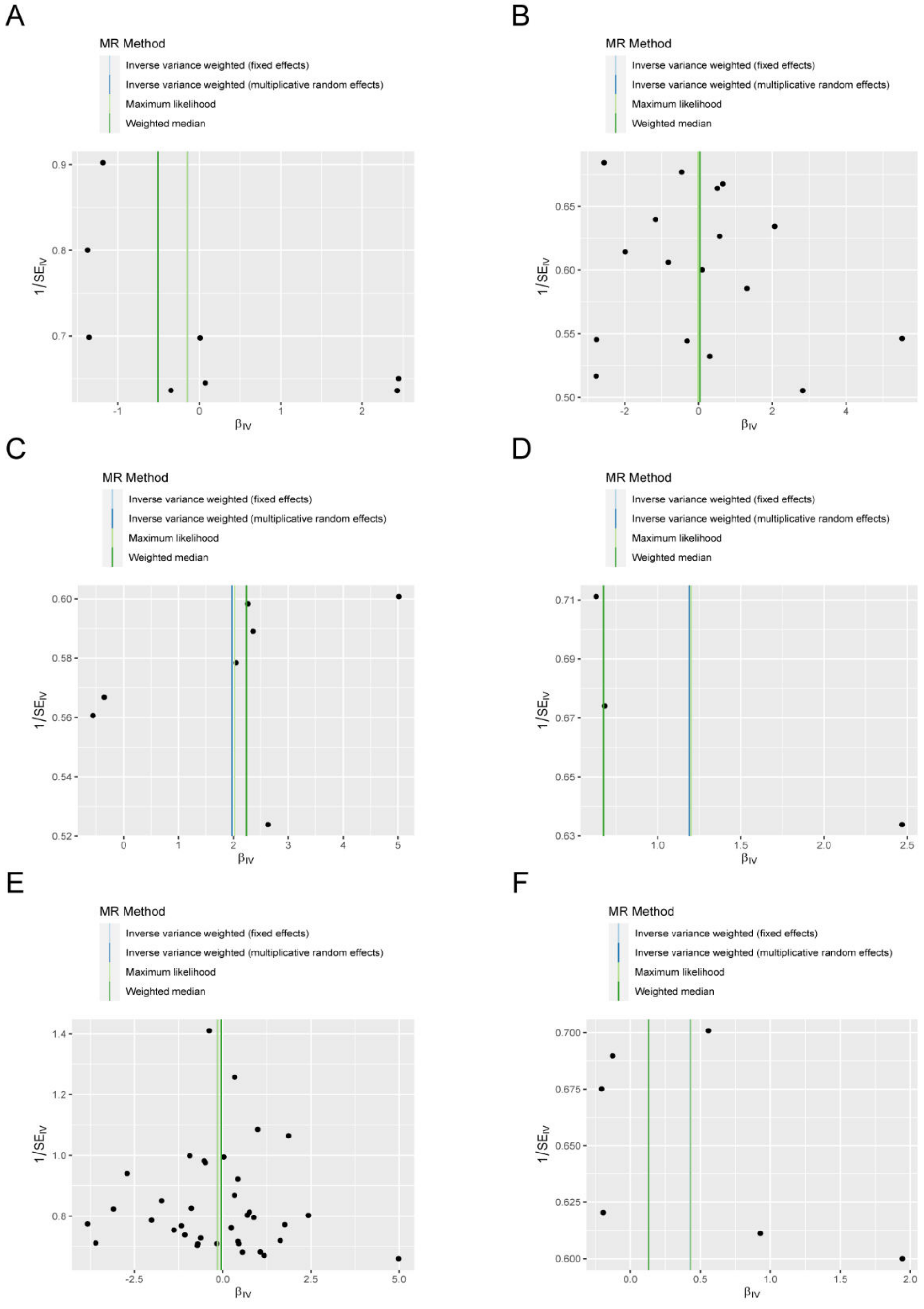

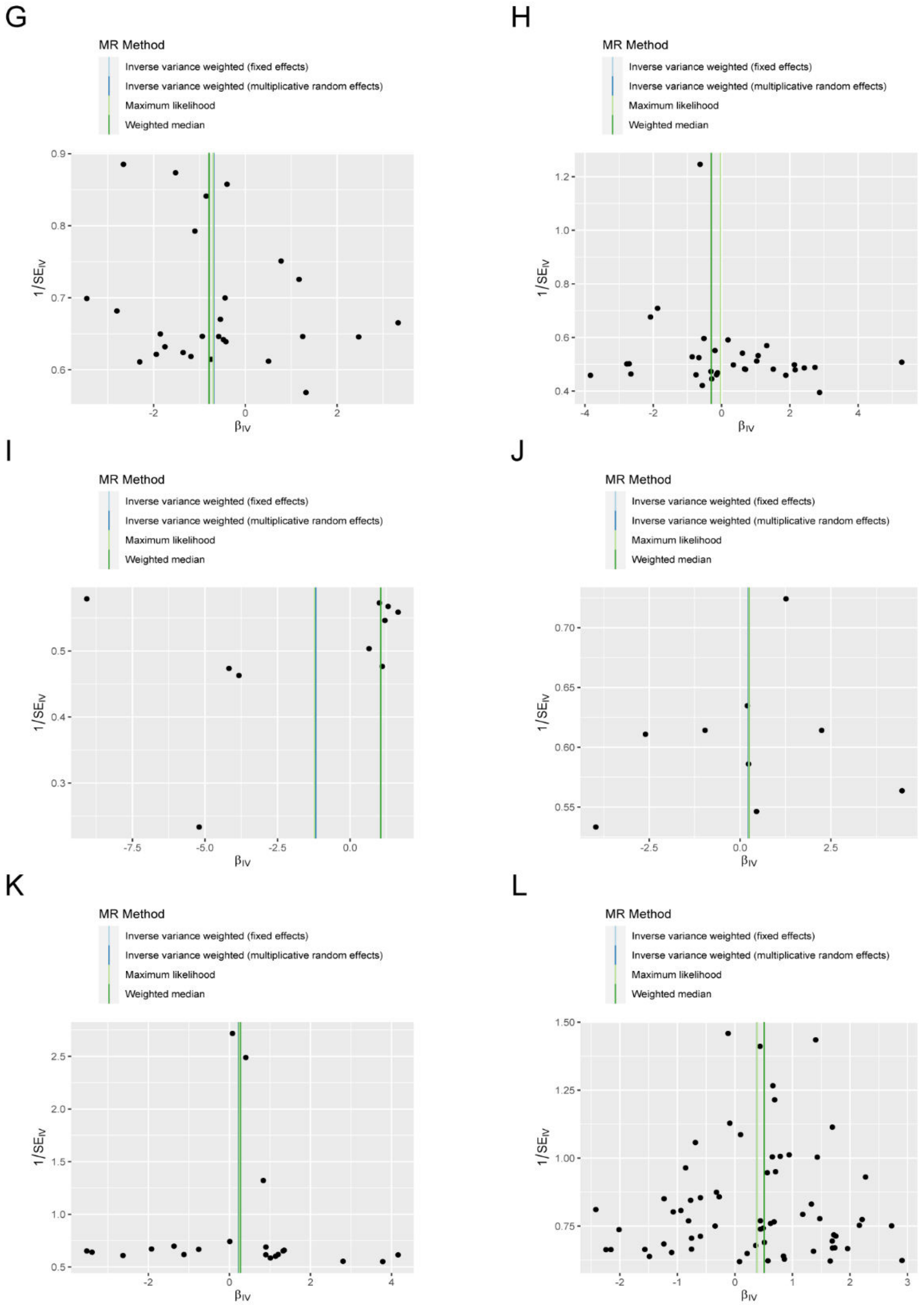
G
MR Method
Inverse variance weighted (fixed effects)
Inverse variance weighted (multiplicative random effects)
Maximum likelihood
Weighted median
1/SE_IV
β_IV
H
MR Method
Inverse variance weighted (fixed effects)
Inverse variance weighted (multiplicative random effects)
Maximum likelihood
Weighted median
1/SE_IV
β_IV
I
MR Method
Inverse variance weighted (fixed effects)
Inverse variance weighted (multiplicative random effects)
Maximum likelihood
Weighted median
1/SE_IV
β_IV
J
MR Method
Inverse variance weighted (fixed effects)
Inverse variance weighted (multiplicative random effects)
Maximum likelihood
Weighted median
1/SE_IV
β_IV
K
MR Method
Inverse variance weighted (fixed effects)
Inverse variance weighted (multiplicative random effects)
Maximum likelihood
Weighted median
1/SE_IV
β_IV
L
MR Method
Inverse variance weighted (fixed effects)
Inverse variance weighted (multiplicative random effects)
Maximum likelihood
Weighted median
1/SE_IV
β_IV
